# Introducing a new measure for assessing self-efficacy in response to air pollution hazards for pregnant women

**DOI:** 10.1186/2052-336X-11-16

**Published:** 2013-07-08

**Authors:** Marzieh Araban, Seddigheh Sadat Tavafian, Saeid Motesaddi Zarandi, Ali Reza Hidarnia, Mahmood Reza Gohari, Janice M Prochaska, Afsaneh Laluie, Ali Montazeri

**Affiliations:** 1 Department of Health Education, Faculty of Medical Sciences, Tarbiat Modares University, Tehran, Iran; 2 Department of Environmental Health Engineering, Faculty of Health, Shaheed Beheshti University of Medical Sciences, Tehran, Iran; 3 Department of Biostatistics, Hospital Management Research Center, School of Health Management and Information Sciences, Iran University of Medical Sciences, Tehran, Iran; 4 Prochange Behavior System, West Kingston, USA; 5 Department of Gynecology, Faculty of Medical Sciences, Baqiyatallah University of Medical Sciences, Tehran, Iran; 6 Mental Health Research Group, Health Metrics Research Center, Iranian Institute for Health Sciences Research, ACECR, Tehran, Iran

**Keywords:** Air polltion, Confirmatory factor analysis, Iran, Pregnant women, Reliability, Self-efficacy, Validity

## Abstract

A self-efficacy instrument should be condition-specific. There are several instruments for measuring self-efficacy, but none are air pollution-specific. This study aimed to develop a self-efficacy measure for assessing pregnant women’s responses to air pollution hazards. A random sample of pregnant women aged between 18 and 35 years attending three prenatal care centers were entered into the study. Prenatal care centers randomly selected from a list of centers located in different geographical regions of Tehran, Iran. After careful consideration and performing content and face validity, a 4-item measure was developed and participants completed the questionnaire. Reliability was estimated using internal consistency and validity was assessed by performing confirmatory factor analysis (CFA) and known group comparison. In all 200 eligible pregnant women were studied. The mean age of participants was 26.9 (SD = 4.8) years and it was 27.9 (SD = 9.1) weeks for gestational age. The findings showed almost perfect results for both content validity ratio (CVR = 1) and content validity index (CVI = 1). The confirmatory factor analysis indicated a good fit to the data, and known group comparison revealed satisfying results. Internal consistency as measured by the Cronbach’s alpha coefficient was found to be 0.74. In general, the findings suggest that this new generated scale is a reliable and valid specific measure of self-efficacy in response to air pollution hazards for pregnant women. However, further studies are needed to establish stronger psychometric properties for the questionnaire.

## Introduction

Globally, it has been estimated that 24% of all disease burden (healthy life years lost) and 23% of all premature deaths were attributable to environmental issues of which air pollution was the most important contributing factor [1]. It is argued that air pollution could cause 3% of mortality due to cardiopulmonary diseases and 5% of mortality due to respiratory system cancers, leading to 800000 premature deaths and 6400000 years of life lost [2]. This feature of air pollution consequences occurs predominantly in developing countries among them Asia is more vulnerable [2].

Tehran, the capital city of Iran, is one of the most polluted cities in the world [3]. Despite applying some policies and regulations in Iran, existed evidence has revealed that the current level of air pollutants in Tehran is above healthy levels. Although, air pollution is very harmful for population health, this hazard could be more severe for higher risk groups such as pregnant women [4,5]. Some adverse outcomes of air pollution for pregnant women include low birth weight, preterm labor, and intrauterine growth retardation that in turn could be leaded to a range of childhood and adulthood morbidity and mortality later [6-8] imposing extra costs to the health system [9]. However, there is a strong believe that we could prevent pregnant women from being exposed to air pollution [3]. As such, the most suggested preventive strategy is to reduce exposure to air pollution by reducing time spent outdoors [10-12].

It is well documented that effective preventive strategies and interventions should be theory-driven or at least should have constructs that might increase the likelihood of behavioral changes toward desired outcomes [13-15]. For instance, self-efficacy is a key construct in many health education theories and models [16]. Those interventions that contained self-efficacy as a basic element showed more satisfying results [17,18]. Self-efficacy is defined as people’s beliefs in their own abilities to perform a given behavior [19]. These perceptions contribute to individuals’ judgments in their own abilities to perform a specific behavior and greatly influence their choice of or maintaining in doing that behavior [20]. As indicated above, it has been consistently shown that self-efficacy is one of the most important predictors of health behaviors [21-24]. Thus we thought it would be important to focus on self-efficacy in order to change exposure behavior among pregnant women.

There are several instruments for measuring self-efficacy [21-32]. Examples of these instruments are presented in Table 1. None of these instruments were air pollution specific. Hence, the aim of this study was to develop a self-efficacy measure for assessing the prevention of air pollution hazards. It was hoped this might help to fill the gaps and perhaps contribute to the existing literature on the topic.

**Table 1:** Examples of Self-efficacy measures used in the literature

**Author(s) [ref.]**	**Year**	**Scale**	**Number of items**	**Response format**
Schwarzer [21]	1995	General Self Efficacy	10	Definitely not to exactly true
Barkley & Burns [22]	2000	Condom Use Self-Efficacy	10	Strongly disagree to strongly agree
Schwarzer & Renner [23]	2000	The Nutrition Self-Efficacy	5	Very uncertain to very certain
Schwarzer & Renner [23]	2000	The physical exercise Self-Efficacy	5	Very uncertain to very certain
Schwarzer & Renner [23]	2000	The Alcohol Resistance Self-Efficacy	3	Very uncertain to very certain
Mohr et al. [24]	2001	Adherence (self injection) self-efficacy	1	I will not have any problems injecting myself to I will not be able to tolerate it at all.
Ma et al. [25]	2002	Consumption of fruit and vegetables Self-Efficacy	5	Not at all confident to very confident
Dennis [26]	2003	Breastfeeding Self-Efficacy Scale-Short Form	14	Not at all confident to 5 always confident
Luszczynska & Schwarzer [27]	2003	Preaction BSE Self-Efficacy Scale:	4	Definitely not to exactly true
Luszczynska & Schwarzer [27]	2003	Maintenance BSE Self-Efficacy Scale	2	Definitely not to exactly true
Kerr et al. [28]	2004	Adherence to therapy self-efficacy	6	Not reported
Kerr et al. [28]	2004	Adherence to therapy regulatory self-efficacy	2	Not reported
Kronborg et al. [29]	2007	Health visitor’s Self-efficacy	5	Five-point Likert scale
Clayman et al. [30]	2010	Patient Communication Self-Efficacy (AURA)	4	A lot disagree to a lot agree
Latimer et al. [31]	2011	Self-efficacy for weight loss (nutrition and exercise)	11	Not at all confident to completely confident
Schwarzer & Luszczynska [32]	2012	Smoking cessation Try Self-Efficacy	5	‘Not at all sure I am able to, to ‘very sure I am able to’

## Materials and methods

### The questionnaire

Self-efficacy provided the theoretical concept for the instrument development. Albert Bandura has defined self-efficacy as one’s belief in his/her ability to succeed behavior changes in specific situations [19]. Thus as indicated earlier (Table 1) and considering several different self-efficacy measures, we produced a set of specific items for this study as recommended by Bandura. His main recommendation relies on the fact that a self-efficacy measure should be condition-specific. In this study the time spent outdoors by pregnant women was defined as a specific situation. Accordingly, decreasing this time was considered as a desired behavior to be adopted by pregnant women. In fact the desired behavior (prevention of air pollution hazards) was defined as any activities resulting in decreased exposure time to air pollution. To be more specific we asked environmental health experts to indicate necessary recommendations for pregnant women. Then the idea of ability to perform the recommended behaviors was set to develop the concept and generate items to provide a measure for self-efficacy. Overall 16 items resulted from the above mentioned approaches. After careful examination and recombination of similar items or items very close in meanings, the following 5 items remained:

1). I can stay indoors in the peak hours of the air pollution - from 7.00 o’clock to 9.00 o’clock in the morning 2). I can stay indoors in the peak hours of the air pollution - from 6.00 o’clock to 9.00 o’clock in the evening; 3). I can stay home in the days that air quality is in the crisis situation; 4). I can avoid entering into the high traffic area of the city. 5). I can wear an air filtering face mask during my walks through the city center. Each item is rated on a 4-point Likert scale ranging from ‘not at all sure’ to ‘completely sure’ giving a possible score of 1 to 4 for each item and 4 to 16 for the total items.

### Sampling

A multi stage cluster sampling was applied. First Tehran was divided into 3 regions: north, center and south. Among all prenatal health care centers located in these three regions, one center was randomly selected. Then from pregnant women attending to the center, a random sample was selected through random numbers. The sample size was estimated on the basis of our planned procedure for confirmatory factor analysis. It has been suggested that a sample size of 100 to 200 individuals is an acceptable sample size if the model is not complex in the confirmatory factor analysis. Thus a sample of 200 women was thought for this study [33]. The inclusion criteria were: being aged 18 to 35 years old, having the history of pregnancy without adverse outcomes, not suffering from chronic diseases during the present pregnancy and not having the history of fertility problems. Demographic characteristics of the pregnant women included recoding of age, education of pregnant women and their husbands, gestational age, and family monthly income.

### Statistical analysis

In this study face, content and construct validity of the designed instrument was performed as follows:

#### *Face validity*

Both qualitative and quantitative methods were applied for face validity. For the purpose of qualitative approach, 20 pregnant women were asked to assess each item for ambiguity and difficulty. In general, there were no problems in reading and understanding the items by pregnant women. The quantitative face validity was evaluated through impact score. The impact score for each item was calculated as multiplying the importance of an item with its frequency. The impact scores of greater than 1.5 were considered suitable [34].

#### *Content validity*

An expert panel including 15 health education, environmental health, obstetrics and maternal child health specialists examined the content validity. The expert panel was asked to comment on the necessity and relevance of the items in order to calculate the Content Validity Ratio (CVR) and the Content Validity Index (CVI), respectively. The necessity of an item was assessed using a three-point rating scale: (i) not essential, (ii) useful, but not essential, (iii) essential. Following the experts’ assessments, the CVR for total scale was computed. According to Lawshe, if more than half of the panelists indicate that an item is essential, then that item has the least content validity [35]. Here, the CVR for the scale equal or greater than 0.59 was considered satisfactory. The CVI was estimated by experts’ ratings of items relevancy, simplicity, and clarity on a 4-point Likert scale. The CVI of each statement was calculated and as recommended values of equal or greater than 0.80 were considered acceptable [36].

#### *Factor structure*

Confirmatory Factor Analysis (CFA) was carried out to test whether the data fit the hypothesized measurement model. Usually for the confirmatory factor analysis 2 to 3 items are enough to carry out the analysis and there are several fit indices for evaluating model fit [33]. We will report on some of the most important fit indexes with their cut of points as follows: Chi-Squared Test Values closer to zero indicate a better fit, For Root Mean Square Error of Approximation, a value of .06 or less is indicative of acceptable model fit. GFI and CFI value of .90 or larger is generally considered to indicate acceptable model fit [33,37].

#### *Discriminant validity*

Discriminant validity of the instrument was assessed using known groups comparison. Known groups comparison was performed to test how well the questionnaire discriminates between women in different stages of behavior change (pre-action stage and action stage).

#### *Reliability*

Internal consistency of the instrument was assessed by using Cronbach’s alpha coefficient. Alpha values of equal or greater than 0.70 was thought satisfactory [38].

### Ethics

The ethics committee of Tarbiat Modares University approved the study. Informed consent was obtained from participants.

## Results

In total, 200 pregnant women completed the questionnaire. The mean age of women was 26.9 (SD = 8.4) years and the mean gestational age was 27.9 (SD = 9.1) weeks. The characteristics of participants and self-efficacy scores by demographic characteristics are shown in Table 2.

**Table 2:** The characteristics of the study sample and self-efficacy scores by demographic status (n = 200)

		**Number (%)**	Self-efficacy *
**Age (years)**			
	18-23	61 (30.5)	13.3 (2.3)
	24-30	85 (42.5)	12.6 (2.1)
	31-35	54 (27)	12.3 (3.3)
*Test result (P-value)*			0.23**
**Gestational age (weeks)**			
	< 12	20 (10)	12.75 (2.6)
	13-28	64 (32)	12.76 (2.1)
	29-36	116 (58)	12.79 (2.8)
*Test result (P-value)*			0.9**
**Parity**			
	Nullipareous	97 (48.5)	12.7 (2.9)
	Multiparous	103 (51.5)	12.8 (2.9)
*Test result (P-value)*			0.78***
**Employment**			
	Housewife	192 (96)	12.9 (2.7)
	Employed	8 (4)	8.3 (3.11)
*Test result (P-value)*			0.001***
**Education**			
	Primary	51 (25.5)	13.2 (3.1)
	Secondary	125 (62.5)	13.5 (2.9)
	Higher	24 (12)	12.5 (2.8)
*Test result (P-value)*			0.14**
**Husband Education**			
	Primary	53 (26.5)	13.3 (3.1)
	Secondary	126 (63)	13.2 (3.1)
	Higher	21 (10.5)	12.5 (2.9)
*Test result (P-value)*			0.25**
**Family income per month**			
	Poor	41 (20.5)	13.6 (3.1)
	Fair	116 (58)	13 (2.8)
	Good	43 (21.5)	12.2 (2.9)
*Test result (P-value)*			0.17**

* Mean (SD). Higher values indicate better self-efficacy.

** Results derived from one-way analysis of variance.

*** Results derived from t-test.

The results obtained from validity analysis showed good levels of the CVR (equal to 1), CVI (equal to 1) and impact score (IS = 5) for four first items. Item 5 was not found as necessary by panelists and it was omitted from further analysis.

**Figure 1:**
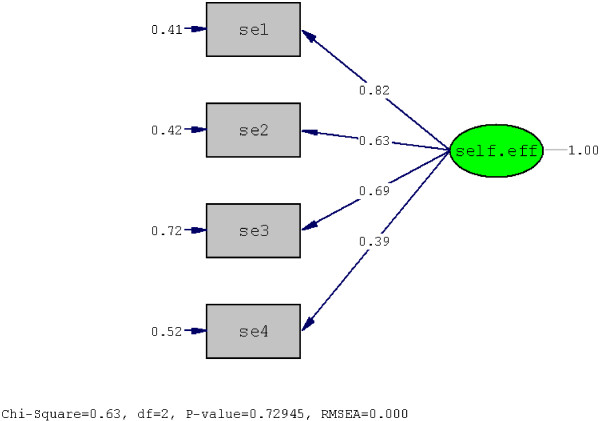
Factor structure of the self-efficacy measure for prevention of air pollution hazards among pregnant women derived from confirmatory factor analysis.

The results from confirmatory factor analysis are shown in Figure 1. Overall all fit indices were found to be satisfactory. The root mean square error of approximation (RMSEA) showed an acceptable value model fit (< 0.0001). The goodness of fit index (GFI) and Adjucted goodness of fit index (AGFI) were acceptable (GFI = 0.97 and AGFI = 0.96).

Validity of the scale as assessed by known groups comparison indicated that the questionnaire discriminated well between sub-groups of women who differed in the stage of behavior change. As expected, those who scored higher on the scale were more likely to be in the action stage (Table 3).

**Table 3:** The descriptive statistics for the self-efficacy measure* (n = 200)

**Item**	**Mean (SD)**	**Possible range**
I can stay indoors in the peak hours of the air pollution - from 7.00 o’clock to 9.00 o’clock in the morning.	3.02 (1.04)	1-4
I can stay indoors in the peak hours of the air pollution - from 6.00 o’clock to 9.00 o’clock in the evening.	3.41 (1.09)	1-4
I can stay home in the days that air quality is in the crisis situation.	2.84 (0.82)	1-4
I can avoid entering into the high traffic area of the city.	3.46 (1.04)	1-4
**Total score**	12.73 (2.92)	4-16
**Cronbach’s alpha coefficient**	0.74	-

* Higher values indicate better self-efficacy.

The result obtained from reliability analysis indicated that alpha coefficient for the scale was 0.74, well above the threshold lending support to its acceptable internal consistency. The results are shown in Table 4.

**Table 4:** Known groups comparison by stages of change*

	**Pre-action**	**Action**	
	**Mean (SD)**	**Mean (SD)**	**P-value**
I can stay indoors in the peak hours of the air pollution - from 7.00 o’clock to 9.00 o’clock in the morning.	2.79 (1.07)	3.52 (0.75)	0.001
I can stay indoors in the peak hours of the air pollution - from 6.00 o’clock to 9.00 o’clock in the evening.	3.24 (0.99)	3.7 (0.48)	0.001
I can stay home in the days that air quality is in the crisis situation.	2.60 (1.1)	3.38 (0.85)	0.001
I can avoid entering into the high traffic area of the city.	3.33 (0.86)	3.74 (0.62)	0.001
**Total score**	11.97 (2.97)	14.44 (1.92)	0.001

* Higher values indicate better self-efficacy.

## Discussion

The findings demonstrated that the air pollution self efficacy measure developed by this study obtained good validity values. In addition we found that the pattern of observed level of self efficacy across the stages of change was consistent with health education theory’s prediction [39]. Self-efficacy was lower among participants who were in pre action stages and was higher among those who were in the action stages. The findings from current study were consistent with previous findings on the topic [16,40-43] indicating that targeting interventions that focus on self-efficacy as a theoretical framework for a desirable behavior (that was reduced time spent outdoors) might lead to acceptance of a behavior (that was prevention of air pollution hazards).

Few studies have investigated the validity of self-efficacy scales using confirmatory factor analysis. In line with our study, Latimer et al. [31] reported a unidimensional scale for weight loss among women with a sedentary lifestyle while Barkley and Burnes [22] reported a three-dimensional tool for condom use self-efficacy. One possible explanation for such differences in construct of these questionnaires is the fact that these tools were used for different types of behaviors. For instance while weight loss is a matter of personal attempt, condom use self-efficacy is a unique behavior that requires interaction between two partners.

As Bandura [19] advocated a behavior-specific approach to the study of self-efficacy, he argues that a measure of general self-efficacy in overall ability for tapping an individual’s efficacy in managing tasks associated with a specific behavior would be inadequate. Thus, to assess air pollution exposure self-efficacy, an instrument specific to tasks could lead to more concise values of self-efficacy as compared to using a general self-efficacy measure as reported by Schwarzer [21].

The validity of the scale was strengthened further by estimates of how well the observed indicators (each item on the measure) served as a measurement tool for the construct of self-efficacy related to the prevention of air pollution hazards behaviors. These estimates all were suitable, providing strong evidence that each item reflected self-efficacy. The Cronbach’s alpha coefficient was 0.74 and seems very satisfying for a 4-item scale. It is argued that using large scales are not necessary for predicting a health behavior and rather using rigorous theory-based item wording is more important than the number of statements of an scale [29]. Thus, one might conclude that our short scale could be useful to measure self-efficacy. Self-efficacy scores are very important for setting priorities when developing an specific intervention. One goal of developing a scale is to construct parsimonious measures that can be integrated into a more comprehensive questionnaire [24]. As such we feel our new scale could be integrated into interventions based on many health education and promotion theories and models such as: TransTheoretical Model, Social Cognitive Theory, Theory of Planned Behavior and Health Belief Model; where all have the construct of self-efficacy and are among the most used theories for health behavior change [16].

In summary, one of the most important millennium development goals is to improve maternal health. Also millennium goal 7 and 8 state that the study of air pollution and its potential public health impact on the general population and highly susceptible groups such as pregnant women should be a priority [44]. In addition it is recommended that one goal of any program is to be measured correctly [45]. Yet, we thought developing a measure of self-efficacy for prevention of air pollution hazards for pregnant women might cover these goals in particular and help to improve women’s health in general.

The current study, however, had some limitations. Almost all participants (96%) were housewives. Perhaps further testing of the measure is needed with employed pregnant women. In addition since our analysis was not based on the maximum required sample size, its replication in a larger sample is warranted to confirm the factor structure of the measure.

## Conclusion

In general, the findings suggest that this new generated scale is a reliable and valid specific measure of self-efficacy in response to air pollution hazards for pregnant women. However, further studies are needed to establish stronger psychometric properties for the questionnaire.

## Competing interests

The authors declare that they no competing interests.

## Authors’ contributions

MA was the main investigator, collected the data, performed the statistical analysis, and drafted the manuscript. SST supervised the study. SMZ and ARH were advisors of the study. MRG helped in statistical analysis. JMP and AL helped as consultants. AM was the supervisor of the study, contributed to analysis, and provided the final article. All authors read and approved the final manuscript.
